# Editorial: Stretch and the heart: mechanoelectrical coupling and arrhythmias

**DOI:** 10.3389/fphys.2023.1278561

**Published:** 2023-08-25

**Authors:** Ewan Douglas Fowler, Jan E. Azarov, Fabien Brette

**Affiliations:** ^1^ School of Biosciences, College of Biomedical and Life Sciences, Cardiff University, Cardiff, United Kingdom; ^2^ Department of Cardiac Physiology, Institute of Physiology, Komi Science Center, Ural Branch, Russian Academy of Sciences, Syktyvkar, Russia; ^3^ Institute of Medicine, Pitirim Sorokin Syktyvkar State University, Syktyvkar, Russia; ^4^ INSERM U1045 -Université de Bordeaux, Bordeaux, France; ^5^ IHU LIRYC-CRCTB U1045, Pessac, France; ^6^ Phymedexp INSERM U1046, CNRS, Université de Montpellier, Montpellier University Hospital (CHRU), Montpellier, France

**Keywords:** stretch, mechanoelectrical coupling, arrhythmias, ion channels, Purkinje fiber, computational modelling

In cardiomyocytes, excitation-contraction coupling, the process that links electrical activity to cell contraction, is well established (Bers, 2002). On the contrary, the mechano-electric feedback (MEF) mechanism that can be summarized by how mechanical stimulation can modulate electrophysiology and mechanical function is less understood (Quinn and Kohl, 2021). This is true at the cellular, tissue and organ level. It is well known that stretch in the heart can cause changes to the electrical activity through MEF, and it has even been suggested that this feedback plays a role in the mechanical initiation of arrhythmias and fibrillation. MEF generally serves for rapid adaptation of cardiac function to changing hemodynamical load. There are several mechanisms that mediate MEF including mechanically gated and mechanically modulated ion channels, mechanosensitive intracellular enzymes, cytoskeleton elements, calcium handling, and more (Quinn and Kohl, 2021). On the other hand, abnormal mechanical stimulation can result in life-threatening arrhythmias via MEF mechanisms in various conditions, such as commotio cordis, acute ischemia, chronic heart failure, etc. Mechanically induced arrhythmogenesis has long been a focus of clinical, experimental and simulation studies.

In this Research Topic, we present six articles devoted to some understudied and emerging aspects concerning MEF mechanisms and significance to heart function.

The Research Topic starts with two research articles on mechanosensitive sarcoplasmic reticulum (SR)-resident channels. It has been known for decades that SR membranes have a high Cl^−^ and K^+^ conductance that act as counter ion channels in the SR membrane to facilitate calcium fluxes (Dulhunty et al., 1996).

Using an emerging animal model for cardiac physiology, Zechini et al. provide evidence that Piezo is an SR-resident channel with an important role in buffering mechanical stress in the *Drosophila* heart. The use of *Drosophila* in cardiac physiology is emerging and promising given its genetic tractability (Zhao et al., 2023). It remains to be investigated whether the same mechanism is present in mammals. Given the high degree of evolutionary conservation of Piezo throughout the animal kingdom, and even all eukaryotic kingdoms (Coste et al., 2010), this raises interesting questions regarding its possible role in the Frank-Starling mechanism.

There is a growing research community investigating SR Ca^2+^ leak channels (Takeshima et al., 2015). Streiff et al. demonstrate that transient receptor potential canonical 1 (TRPC1) channels form a mechano-modulated SR Ca^2+^ leak channel in neonatal rat ventricular myocytes. TRPC1 was first described as a Ca^2+^ leak channel in skeletal muscle (Berbey et al., 2009). In this Research Topic, Streiff et al. establish that TRPC colocalize with sarco/endoplasmic reticulum Ca^2+^ ATPase. Using a clever approach to stretch myocytes (stretchable silicone membranes), they show stretched TRPC1-overexpressing neonatal rat ventricular myocytes exhibited a decrease in SR Ca^2+^ load compared to control.

Given the technical challenge to stretch single intact cardiac myocytes, for example, using carbon fibers (Le Guennec et al., 1990), studies at the tissue and organ level are useful and allow the study of interactions between cell types. Purkinje fibres are a known culprit for ventricular arrhythmia (Haissaguerre et al., 2016). They have a distinct cellular electrophysiology from the adjacent endocardium, including longer action potential duration, faster electrical conduction and different Ca^2+^ handling mechanisms that can predispose to ectopic after depolarizations. Retrograde conduction through the Purkinje fibre network can also occur due to its unique anatomical relationship with the greater mass of ventricular myocardium that provides a favourable source-sink balance for ectopic foci in the Purkinje-myocardial junction to trigger action potentials in Purkinje fibres (Blackwell et al., 2022). Consequently, Purkinje fibres have been a target for ablation therapy, however their role in stretch-mediated arrhythmias is less well understood. In this Research Topic, Hurley et al. used a perfused rabbit heart model to investigate the role of Purkinje fibers in acute stretch/dilation-induced ectopics. Acute stretch was imposed by inflating an indwelling balloon in the left ventricle, which normally provoked ectopic beats, but these were prevented when the Purkinje fibre network was preferentially ablated (Haissaguerre et al., 2016). Hurley et al. concluded that the Purkinje fiber network can mediate stretch-induced ectopic beats.

In a review article, Bechard et al. discuss the possible role played by TREK-1 in physiological and pathophysiological conditions and its potential role in MEF since the channel can be mechano-activated. This two-pore-domain potassium channel, first cloned in 1996 (Fink et al., 1996) produce a background current that oppose membrane depolarization and cell excitability. Bechard et al. propose that the presence of TREK-1 would compensate the inward stretch-activated current, especially in stiffer cells.

Two computational modelling papers emphasizing understudied aspects of MEF.

The potential role of non-myocytes (such as fibroblasts, macrophages, or intracardiac neurons) in MEF responses is just emerging, and much remains to be explored. Due to technical challenge at the experimental level, computational models are crucial to disentangle interacting feedback pathways. Kursanov et al. demonstrated that electromechanical interaction with myocardial fibroblasts increased susceptibility of cardiomyocytes to triggered activity in calcium overload. This effect was due to the activation of the mechanosensitive channels of fibroblasts, which caused depolarization of the resting membrane potential in fibroblasts, which leads to corresponding resting membrane depolarization in the coupled cardiomyocytes. It reduced repolarization reserve in cardiomyocytes, which protects from afterdepolarizations, and therefore the fibroblast-cardiomyocyte mechanical interaction renders the cardiomyocytes more susceptible to arrhythmic stimuli. The studied effect also emphasizes that the role of fibroblasts should not be ignored when predicting the effects of mechanical interventions on the heart.

In a second paper, Dokuchaev et al. evaluated importance of mechanical testing for the population of *in silico* models to be used for drug testing. They generated a population of electrophysiological models, which were calibrated using human myocardial force characteristics and mechanical tests involving variations in preload and afterload. Models that passed the mechanical tests were validated with additional experimental data, including the effects of drugs with high or low pro-arrhythmic risk. The authors showed that some models that demonstrated normal action potentials and Ca^2+^ transients with acceptable characteristics may reveal anomalies in the mechanical tests. This study highlights the importance of mechanical testing even in settings where only electrical abnormalities are considered, and only ionic mechanisms are involved.

In conclusion, this Research Topic is covering the broad range of mechanoelectrical coupling in the heart at different levels. Clearly more basic, translational and clinical research is needed to delineate the complexity of MEF. New experimental approaches, for example, at the cellular level the patch-clamp-in-gel technology (Hegyi et al., 2021) should provide more comprehensive understanding of the mechano-transduction mechanisms in cardiomyocytes. New *in vivo* experimental interventions in the intact human heart provide new opportunities to translate and validate preclinical and computer modelling findings, e.g., (Orini et al., 2021). We hope that it encourages further investigation of these exciting areas.
